# Modeling and Validation of Oocyte Mechanical Behavior Using AFM Measurement and Multiphysics Simulation

**DOI:** 10.3390/s25175479

**Published:** 2025-09-03

**Authors:** Yue Du, Yu Cai, Zhanli Yang, Ke Gao, Mingzhu Sun, Xin Zhao

**Affiliations:** 1 School of Computer and Information Science, Qinghai Institute of Technology, Xining 810016, China; ydu@qhit.edu.cn; 2 National Key Laboratory of Intelligent Tracking and Forecasting for Infectious Diseases, The Engineering Research Center of Trusted Behavior Intelligence, Ministry of Education, The Tianjin Key Laboratory of intelligent Robotics (tiKLIR), Institute of Robotics and Automatic Information System (IRAIS), College of Artificial Intelligence, Nankai University, Tianjin 300350, China; 2120220545@mail.nankai.edu.cn (Y.C.); 2120230594@mail.nankai.edu.cn (Z.Y.); 2120240587@mail.nankai.edu.cn (K.G.); sunmz@nankai.edu.cn (M.S.); 3 Institute of Intelligence Technology and Robotic Systems, Shenzhen Research Institute of Nankai University, Shenzhen 518083, China

**Keywords:** cell mechanical model, viscoelastic properties, AFM, creep behavior, layered structure, three-phase flow model

## Abstract

Mechanical models are capable of simulating the deformation and stress distribution of oocytes under external forces, thereby providing insights into the underlying mechanisms of intracellular mechanical responses. Interactions with micromanipulation tools involve forces like compression and punction, which are effectively analyzed using principles of solid mechanics. Alternatively, fluid–structure interactions, such as shear stress at fluid junctions or pressure gradients within microchannels, are best described by a multiphase flow model. Developing the two models instead of a single comprehensive model is necessary due to the distinct nature of cell–tool interactions and cell–fluid interactions. In this study, we developed a finite element (FE) model of porcine oocytes that accounts for the viscoelastic properties of the zona pellucida (ZP) and cytoplasm for the case when the oocytes interacted with a micromanipulation tool. Atomic force microscopy (AFM) was employed to measure the Young’s modulus and creep behavior of these subcellular components that were incorporated into the FE model. When the oocyte was solely interacting with the fluids, we simulated oocyte deformation in microfluidic channels by modeling the oocyte-culture-medium system as a three-phase flow, considering the non-Newtonian behavior of the oocyte’s components. Our results show that the Young’s modulus of the ZP and cytoplasm were determined to be 7 kPa and 1.55 kPa, respectively, highlighting the differences in the mechanical properties between these subcomponents. Using the developed layered FE model, we accurately simulated oocyte deformation during their passage through a narrow-necked micropipette, with a deformation error of approximately 5.2% compared to experimental results. Using the three-phase flow model, we effectively simulated oocyte deformation in microfluidic channels under various pressures, validating the model’s efficacy through close agreement with experimental observations. This work significantly contributes to assessing oocyte quality and serves as a valuable tool for advancing cell mechanics studies.

## 1. Introduction

The global in vitro fertilization (IVF) market has been expanding rapidly over recent years, underscoring the economic and clinical importance of innovations aimed at improving oocyte quality assessment. Valued at approximately USD 26.3 billion in 2024, the market is projected to reach USD 41.9 billion by 2033, with a compound annual growth rate of around 5.3%. The mechanical properties of oocytes are reliable indicators for assessing oocyte quality. Given the characteristic changes in the viscoelasticity of oocytes and early embryos during physiological processes, it is entirely reasonable to speculate that the health status of oocytes and embryos is closely related to their mechanical properties. During oocyte maturation and fertilization, the viscoelasticity of the zona pellucida (ZP) undergoes characteristic changes as the oocyte progresses through different meiotic stages to eventually develop into a fertilized egg [1]. The ZP softens during maturation to facilitate sperm penetration [2,3]. In contrast, the ZP hardens due to the cortical reaction, preventing polyspermy during fertilization. In addition, the thickness of the ZP is closely associated with the reproductive competence of oocytes. A thinner ZP is significantly correlated with low fertilization rates or blastocyst formation rates. The viscosity of the cytoplasm changes during follicular development and maturation. During ovulation, the cytoplasm transitions from an aqueous to a more viscous substance. Throughout maturation, the viscosity of the cytoplasm increases and its mechanical properties exhibit a transition from soft glassy to viscous substance over time scales ranging from less than 1 ms to 1 s [4]. It has been observed that changes in the viscosity of second meiotic oocytes suggest that the viscosity can serve as a significant factor for assessing the quality of mature oocytes. The size and persistence of the injection funnel after intracytoplasmic sperm injection have proven to be indicators of cytoplasm viscosity, with larger and more persistent funnels suggesting greater cytoplasmic viscosity. Studies have found that a higher viscosity or persistent injection funnel are prognostic indicators of poor embryonic development [5]. The aforementioned studies indicate that changes in the mechanical properties of the subcellular components of oocytes are highly correlated with oocyte quality. The in situ measurement of the mechanical properties of oocyte components can serve as an indicator for assessing oocyte quality.

The mechanical models of cells help to further understand the mechanical properties of cells, including the mechanisms of intracellular mechanical responses and of changes in cell morphology. This understanding is crucial for studying processes such as cell migration, division, and differentiation [6,7,8,9]. Wintner et al. [10] compared the effectiveness of the Maxwell, Kelvin, and Burgers models in simulating the response of the cell nucleus under suction. The experimental results showed that the Burgers model achieved the highest R-square goodness of fit values when simulating the creep compliance curve of the nucleus. Massou et al. [11] described cells as a mechanical transducer composed of several elements (talin, integrin, and actin), displaying the trapezoidal displacement with hysteresis under stress. Shao et al. [12] developed a computational model based on the phase-field method to describe cell morphology dynamics. This model considered membrane bending forces, surface tension, surface constraints, and a network of cross-linked actin fibers. Cell mechanical models are also used to study cell interactions with their microenvironment, including how cells sense and respond to substrate stiffness and shear stress [13]. In Ref. [14], the response of the Lamina was described using an elastic element and a damping element, showing that the stiffness of the cell nucleus changes with the ratio of Lamina A to Lamina B during cell aspiration experiments. Muller et al. [15] utilized a hyperelastic Mooney–Rivlin model to study cell deformation in three-dimensional fluid environments. The accuracy of this model was determined by its three parameters, which were crucial for understanding cell behavior in tissue deformation and regeneration. Yanez et al. [3] constructed a modified Zener model to simulate embryos, which added a viscous element to the original Zener model. This study examined the correlation between the key parameters of the Zener models and blastocyst formation. Although continuum mechanics models can reflect certain mechanical properties of cells, such as viscoelasticity and stiffness, they neglect the mechanical components of cells themselves [16]; however, the mechanical performance of cells is closely related to subcellular components within cells [17].

To address the limitations of treating oocytes as homogeneous entities, recent studies have developed bilayer models that consider the distinct mechanical properties of the ZP and the cytoplasm. These models recognize the layered structure of the oocyte, providing a more accurate representation of its mechanical behavior under various conditions. Shen et al. [18] approximated the oocyte as a layered spherical body composed of the cytoplasm and the zona. The zona was modeled as synthetic polymers with metal–ligand cross-links synthesized and engineered to contain static and dynamic networks, while the cytoplasm was modeled as a dynamic network. Dittmann et al. [19] modeled the zona as an isotropic, highly compressible material with properties given by the strain energy function, and the cytoplasm as a static fluid structure. The material parameters were identified by a forward finite element analysis and an optimization algorithm taking into account the force–strain relations and geometric information. In Ref. [20], the porcine ZP membrane was modeled as a visco-hyperelastic material following the Arruda–Boyce constitutive law. The visco-hyperelastic parameters of the ZP were determined using a hybrid characterization framework combining AFM, microscopy nanoindentation measurements, nonlinear finite element analysis, and nonlinear optimization [21,22]. Tan and Sun [23] used a “cortex-layer liquid-core” model to study the response of cells during microinjection. Several constitutive materials were proposed to model the membrane of embryos, including Mooney–Rivlin, neo-Hookean, and Yeoh. The parameters were adjusted to ensure the results agreed with the experimental observations. Unlike previous studies, this paper presents a novel and direct method for determining the mechanical property parameters of the ZP and cytoplasm in biomechanical models, based on AFM measurements. Traditional approaches often rely on solving complex optimization algorithms, which are computationally intensive and prone to convergence issues due to the high dimensionality and nonlinearity of the problem. In contrast, our method bypasses these inherent challenges by establishing an explicit relationship between the AFM force–indentation data and the mechanical parameters of the biological materials. This significantly simplifies the parameter identification process, making it more efficient and accessible for practical applications. The proposed approach has been rigorously validated through a series of experiments using narrow-necked micropipettes, which provide high-precision deformation measurements and serve as an independent means of verification. The consistency between the experimental data and the model predictions demonstrates the accuracy and robustness of this method.

In addition to layered models, multiphase flow models have been introduced to simulate the interactions between cells and their surrounding fluids, especially within microfluidic systems. These models treat the cells and the culture medium as separate phases, allowing for the analysis of complex fluid–structure interactions. In Ref. [24], a multiphase flow model was used to simulate mechanical interactions between flow and particles (cells) for the cells-on-a-chip devices, in which flow was considered as the fluid phase and particles (cells) were considered as the discrete phase. Lyras et al. [25] employed a Eulerian–Eulerian two-fluid model which regarded RBCs as suspended particles dispersed in plasma. In Ref. [26], the continuous fluids 1 and 2 were added to form a “liquid in liquid” encapsulated structure. Choi et al. [27] proposed a microfluidic approach to enable the encapsulation of mammalian cells through the use of double emulsion drops with an ultra-thin oil shell as the sacrificial template. Unlike the aforementioned studies, this work presents a novel approach by modeling oocytes as a “liquid in liquid” encapsulated structure, which forms a three-phase flow system in conjunction with the culture medium. The rationale for modeling oocytes as a two-phase flow in this study primarily stems from their complex internal structure and dynamic behavior. Oocytes contain various organelles and cytoplasm, which possess different physical properties compared to the ZP, constituting a separate “liquid phase” distinct from the ZP. This internal separation bestows the cell with a two-phase characteristic, resembling a “liquid in liquid” system. A three-phase flow model, incorporating the two-phase oocyte and the surrounding culture medium, can more accurately describe and predict these phenomena. Additionally, a key feature of this study is that the cellular parameters used in the two-phase flow model are directly derived from AFM measurements, providing reliable input data that enhance the applicability of the two-phase flow model. Finally, the three-phase flow model not only captures the structural characteristics of cells under static conditions but also dynamically simulates their responses to stimuli from the environment.

In this study, we developed two distinct viscoelastic models of oocytes to accurately simulate their mechanical behavior under different interaction scenarios. The first model employs a layered solid FE approach to represent oocytes interacting with micromanipulation tools, capturing forces such as compression, squeezing, and puncturing, which are best described by solid mechanics principles. This model allows for a detailed analysis of stress and strain distributions across the oocyte’s layered structures, thereby providing insights into the mechanical responses at the subcellular level. The second model utilizes a three-phase flow framework to describe oocyte interactions exclusively within fluidic environments, such as those encountered in microfluidic devices or culture media. By accounting for fluid–structure interactions, including shear stress at fluid junctions and pressure gradients along microchannels, this model captures phenomena that solid mechanics alone cannot simulate. Together, these complementary modeling strategies enable a comprehensive understanding of oocyte mechanics under both tool-mediated and fluid-mediated conditions, offering a robust platform for predicting oocyte responses in various experimental and physiological settings.

Firstly, we developed a layered solid viscoelastic model of the oocytes with the material parameters for each layer being derived from AFM measurements including the Young’s modulus and creep behavior of these subcellular components. From the creep characteristic curve, the Young’s modulus of each time point was obtained and converted to the shear modulus and imported into ANSYS’s (ANSYS 9.0) Shear Data module for the determination of viscoelasticity. This model was subsequently validated using narrow-necked micropipette experiments. Secondly, we established a three-phase flow model in which the oocyte was represented as a “liquid in liquid” system with the parameters being derived from AFM measurements. The Reynolds number and the flow velocity of the culture medium were determined according to the pipe diameter, the dynamic viscosity of the fluid, and the pressure loss over the length of the pipe. In our simulations, we modeled the deformation of oocytes subjected to shear stress at fluid junctions and compared it with observations from actual experiments. The results showed a high degree of agreement between the simulations and the experimental observations, demonstrating the effectiveness of our models.

The remainder of this paper is organized as follows. Section 2 provides a comprehensive overview of the materials and methodologies employed, including the AFM system used to quantify the viscoelastic characteristics of the ZP and cytoplasm. It further elaborates on the development of a multi-layer finite element model representing the oocyte’s structural composition under mechanical interaction with micromanipulation instruments, as well as a three-phase flow model capturing oocyte medium dynamics in microfluidic environments. Section 3 presents the experimental and simulation results, specifically: (i) the proposed solid-layered finite element model yielded accurate predictions of oocyte deformation during micropipette aspiration, with a mean error of 5.2% compared to empirical observations; and (ii) the three-phase fluid–structure interaction model demonstrated robust predictive capabilities in simulating pressure-dependent oocyte deformation within microfluidic channels, closely aligning with experimental data. Section 4 and Section 5 provide an in-depth discussion and summarize the key conclusions.

## 2. Materials and Methods

### 2.1. AFM Experiments

AFM measurements were carried out immediately upon the arrival of oocytes at the laboratory. We used ovaries acquired from a slaughterhouse and delivered to our laboratory within 2 h of collection in a thermos flask containing sterile saline at 35–37 °C. The experiment was reviewed and approved by the Animal Care and Use Committee of Nankai University on 15 March 2015. Porcine cumulus–oocyte complexes (COCs, 3 to 6 mm) were collected by aspiration into a culture tube and washed three times in an oocyte washing medium (Genmed Scientifics Inc., Wilmington, DE, USA). The COCs were denuded of cumulus cells by pipetting through a fine bore pipette of appropriate diameter following 1 min of exposure to a hyaluronidase solution.

**Figure 1:**
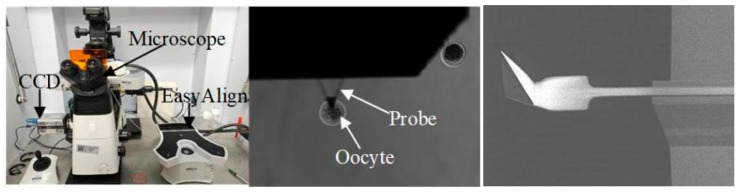
The AFM experimental setup. The Bruker Bioscope Resolve AFM integrates several components, including a baseplate, CCD camera, inverted optical microscope, probe holder, scanning probe microscopy head, and EasyAlign unitI (indentations were performed at the multiple sites on an oocyte).

The AFM instrument we used was a Bruker Resolve (Figure 1), which is specially designed for biological samples. It operated in PeakForce Quantitative Nanomechanics (QNM) fluid imaging mode, where the probe advanced while continuously oscillating. This mode directly controlled the peak force applied by the probe to minimize lateral interference forces, thereby ensuring more precise measurements of the sample’s mechanical response. By limiting the maximum normal load, the system effectively reduced undesired shear or tangential interactions, which are common sources of measurement artifacts in sensitive biological specimens such as soft tissues. In PeakForce QNM mode, the peak force applied by the probe is directly controlled via setting parameters in the software, such as defining the target peak force value. The system uses feedback loops to adjust the cantilever’s deflection, ensuring the actual peak force matches the set value during scanning for precise nanomechanical measurements.

For measuring the Young’s modulus and creep behavior of the ZP, the MLCT-Bio-A probe was utilized. This probe has a triangular geometry, providing better immobilization of porcine oocytes during measurement. The probe specifications are as follows: a tip curvature of 20 nm, a tip length of 4 μm, an overall probe length of 175 μm, a width of 22 μm, a resonant frequency of 22 kHz, and a spring constant of 0.07 N/m. For measuring the Young’s modulus and creep behavior of the cytoplasm, the PFQNM-LC-A-CAL probe with a rectangular geometry was employed due to its high sensitivity and stability. This specific probe design facilitates more effective penetration into porcine oocytes, enabling accurate intracellular measurements by minimizing damage to the cellular membrane and ensuring consistent contact with the cytoplasmic material during indentation testing. When measuring the mechanical properties of soft biological materials (such as cells, extracellular matrices, or hydrogels), spherical, pyramidal, and blunt-cut AFM probes can yield highly consistent elastic modulus results as long as experimental conditions are strictly controlled. Previous studies have shown that when these types of probes are used under the same force–distance curve mode, with identical indentation depths and the same loading/unloading rates, the influence of probe geometry on the calculated modulus can be considered negligible [28,29]. Due to the highly viscoelastic nature of oocytes, which can lead to significant surface deformation prior to achieving meaningful mechanical contact, the engage setpoint for this experiment was carefully set to 0.8 V. This value was chosen to prevent false engagement events that might occur if the probe only indented the soft outer layers without establishing stable contact with the underlying cytoplasmic structure. During the approach phase, once the probe tip made contact with the oocyte surface and the cantilever bending signal reached the predefined threshold of 0.8 V, it was considered a reliable indication of successful engagement. This controlled approach ensured that the initial indentation was both reproducible and minimally invasive, allowing the subsequent mechanical characterization such as force-indentation or creep measurements to be performed under consistent and biologically relevant conditions.

### 2.2. The Viscoelastic Properties of Subcellular Components

To quantify the Young’s modulus of subcellular components within the oocyte, a systematic nanoindentation protocol was employed. An initial measurement site was selected within the central oocyte region to ensure representative sampling. A force–indentation curve was acquired at this location, after which the probe was retracted and repositioned laterally by 2 μm in both the X and Y directions to target adjacent areas. The indentation procedure was repeated at a minimum of five distinct points to account for local heterogeneity in mechanical properties. The resulting force–distance curves were analyzed, and the averaged modulus values were calculated to represent the elastic behavior of the targeted subcellular structures. To measure the Young’s modulus of the cytoplasm in situ, we used adhesive coverslips with stronger adhesion. This slide is a polylysine-coated slide, which mainly utilizes the positive charge property of polylysine to enhance the adhesion of the slide to negatively charged biological samples through electrostatic adsorption. Due to the relatively thick ZP, direct engagement often led to cell slippage. Therefore, we employed a pre-indentation method by recording the height of the probe when measuring the Young’s modulus of the ZP. We manually lowered the probe to the level it reached during the ZP measurement and then increased the setpoint value to 0.9 V to ensure the probe penetrated through the ZP. The force–indentation curve obtained from the cytoplasmic region exhibited a distinct profile compared to that of the ZP. Specifically, the force trajectory recorded by the AFM during indentation displayed a non-monotonic pattern characterized by an initial increase, followed by a decrease, and a subsequent secondary increase, with notably different slopes during the two ascending phases. This behavior strongly suggests that the probe initially penetrated the ZP and subsequently engaged with the underlying cytoplasm in situ. Given that the indentation process involved simultaneous interaction with both the ZP and cytoplasmic layers, the second rising segment of the force curve reflects the mechanical response resulting from the superimposed elastic properties of these two distinct subcellular components. For the fitting model of the tip indentation, compared with most materials, the Young’s modulus of living cells is extremely small, so the influence of the tip curvature radius can be ignored. Therefore, the process of measuring the Young’s modulus of cells by AFM probe indentation can be approximately fitted by the Sneddon model for conical indentation:

(1) $$F=\frac{2}{\pi }\frac{E}{\left(1-v^{2}\right)}\tan\left(\alpha \right)\delta^{2}$$

where E denotes the Young’s modulus, v=0.5 is Poisson’s ratio for the ZP or cytoplasm, and $\alpha =10^{\circ }$ is the half angle of the tip.

For measuring the viscoelastic properties of the ZP, we also used the ramp mode in PeakQNM in a fluid and the Surface Control Groups function to control the probe staying on the sample. After the probe contacted the sample surface and deflected to reach the predetermined force, the same indentation force was maintained, and simultaneously, the variation in the height of the scanning head was recorded to obtain the creep curve of the ZP. The indenting force was maintained for 3, 6, and 10 s. A duration of 6 s was found to be sufficient to fully reflect the creep behavior. From the creep characteristic curve obtained at each indentation point, the corresponding Young’s modulus values were extracted using the Sneddon model. These values were then converted into shear moduli based on established elastic relationships. The resulting shear modulus data were subsequently imported into the Shear Data module of ANSYS to define the material’s time-dependent viscoelastic properties, enabling accurate simulation of the oocyte’s mechanical behavior under varying loading conditions. The “Shear data module” outputs viscoelasticity information mainly because shear tests are highly relevant to the viscoelastic properties of materials. Viscoelastic materials exhibit characteristics of both elasticity and viscosity, meaning their mechanical responses are time-dependent. Shear tests can effectively capture these time-dependent mechanical responses. For example, in a shear creep test, the material is subjected to a constant shear stress, and the resulting shear strain is measured over time. The creep compliance, which is the ratio of shear strain to shear stress, can be used to characterize the viscoelastic behavior of the material. After successfully penetrating the ZP with the probe and obtaining the Young’s modulus of cytoplasm, the probe was retracted while maintaining its relative position with the cell. The setpoint value for engagement was then increased by 0.1 V to ensure penetration into the cytoplasm again. Upon reaching the setpoint value, the change in height of the scanning head was recorded to obtain a force–indentation curve depicting the creep process of the cytoplasm.

(2) $$G=\frac{E}{2(1+\nu )}$$

where G denotes the shear modulus.

### 2.3. Modelling of Oocyte as a Solid-Layered Structure

**Figure 2:**
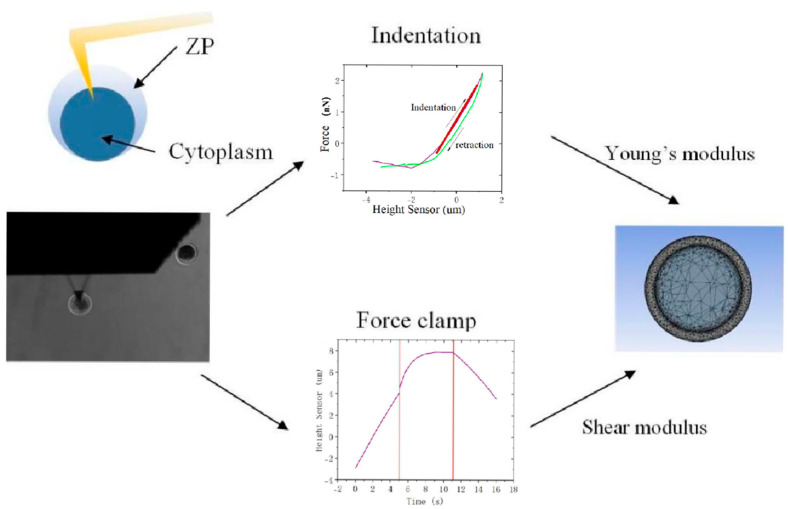
The layered FE model of oocytes. It consisted of a sliding ZP layer and a cytoplasm layer. The shear modulus data and their corresponding tip heights were imported into the Shear Data module of ANSYS software to fit the curves and determine the viscoelastic properties of the cells.

When the oocytes physically interacted with a manipulation tool, we modeled them as a solid-layered structure. According to the experimental observations, the creep data of cytoplasm exhibits shear-thinning non-Newtonian fluid behavior (the measured viscosity decreases from 969.1 Pa s at low strain rates to 44.1 Pa s at high strain rates). To account for the differing mechanical properties of the ZP and the cytoplasm, we established a finite element model with a layered configuration. In this model, the ZP layer and the cytoplasm layer were allowed to slide over each other, with their mechanical properties obtained through AFM measurements. First, we recorded the force–indentation curves for the ZP and the cytoplasm and performed curve fitting using the Sneddon model to obtain the Young’s modulus of these two components. Then, upon reaching predetermined indenting forces, we applied force clamping and recorded the force–indentation curves as creep characteristic curves. After exporting the tip-height data for each point from the curves, we calculated the Young’s modulus at each measurement point and converted it to shear modulus. Subsequently, we imported the shear modulus data and their corresponding tip heights into the Shear Data module of ANSYS software to fit the curves and determine the viscoelastic properties of the cells. The process of building the solid-layered oocyte model is shown in Figure 2. The model consists of inseparable and sliding layers of the ZP and cytoplasm.

### 2.4. Modelling of Oocyte Culture Medium as a Three-Phase Flow

Experiments have shown that with the increase in strain rate, the viscosity decreases significantly and the fluid properties are enhanced. We considered cell deformation at the fluid junction in a microfluidic device. The first step was to determine the flow velocity of the culture medium within the microfluid channel. To achieve this, we calculated the Reynolds number using the equation Re=ρvd/μ, where ρ is density, v is the flow velocity, d is the pipe diameter, and μ is the dynamic viscosity of the fluid. The Reynolds number is a dimensionless number in fluid mechanics, which is used to describe whether the fluid flow is laminar or turbulent. The pressure was 300 hPa, and the diameter of the microfluidic channel was 250 μm. The density of the culture medium was 1008 kg/m^3^, and the dynamic viscosity of the culture medium was $1.66\times 10^{-3}$ Pa s. The high-speed camera operated at a frame rate of 2000 frames per second. After the liquid entered the channel, the movement speed of the culture medium was obtained by measuring the distance the liquid advanced between the two frames. Given the abovementioned parameters, the Reynolds number was calculated to be 106.5. When the Reynolds number was less than 2000, the fluid flow in a straight circular pipe of constant cross-section was laminar flow. When the pressure was 200, 300, 400, 500, 600, 700, or 900 hPa, the fluid flow inside the pipe followed Poiseuille flow. Based on the Poiseuille flow equation $v=-\frac{\Delta p}{4\mu L}(R^{2}-r^{2})$, the fluid velocity could be obtained, where Δp is the pressure loss over the length of the pipe, R is the diameter of the pipe, and r is the distance from the particle to the centerline of the pipe.

Because oocytes exhibit strong viscoelasticity, which displays both the mechanical properties of solids and fluid-like behavior, we considered the ZP and cytoplasm as two shear-thinning non-Newtonian fluids. The entire simulation consists of three fluids: the two non-Newtonian fluids (ZP and cytoplasm) and one Newtonian fluid (cell culture medium). When performing three-phase flow simulations in Fluent, the material’s zero-shear viscosity and infinite-shear viscosity were required. In MATLAB R2024a, the ‘lsqcurvefit’ function is used to perform least-squares fitting on the Carreau model, optimizing parameters to minimize the error between model predictions and experimental data. By fitting the obtained viscosity values of cytoplasm at different shear rates, we derived the parameters for the Carreau model.

## 3. Results

### 3.1. The Mechanical Characterization of Subcomponents of Oocyte

**Figure 3:**
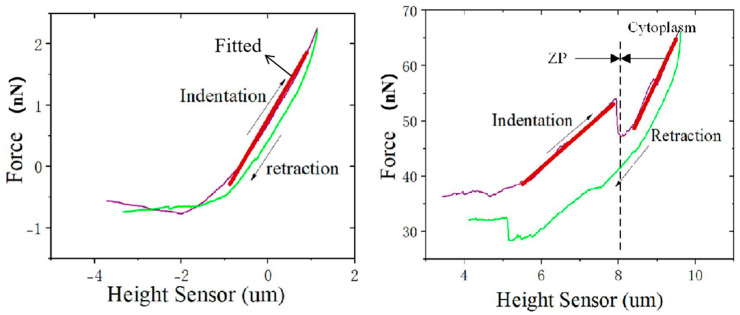
The indentation–retraction curve of the ZP (**left**) and cytoplasm (**right**). The height sensor refers to the piezo displacement. When measuring the mechanical properties of the oocyte’s zona pellucida and cytoplasm using Atomic Force Microscopy (AFM), we employed probes with different tip lengths. The probe with a 4 μm long tip was used for measuring the mechanical properties of the zona pellucida (**left**). For the cytoplasm measurement, we used a probe with an 18 μm long tip (**right**).

We measured the Young’s modulus of the ZP through an AFM indentation experiment. Gradually lowering the probe above the oocyte, the cantilever beam began to bend and the indenting force also began to increase once the probe contacted the cell surface, as shown in Figure 3 left. When the preset force value was reached, the probe began to retract. After multiple measurements on each oocyte, the Young’s modulus of the ZP was determined to be 7.01±2.88 kPa. The loading and unloading force curves did not overlap, which is indicative of time-dependent mechanical behavior such as viscoelasticity, although other effects (e.g., poroelasticity, plastic deformation, or adhesion) may also contribute to the observed hysteresis.

The Young’s modulus of the cytoplasm was subsequently measured using the same AFM-based indentation protocol. Unlike conventional approaches that primarily focus on the initial portion of the force–displacement curve, our method emphasized the curve morphology and fitted parameters following the probe’s penetration into the sample. As illustrated in Figure 3 right, the force curve displays a characteristic profile: an initial increase in force, followed by a transient decrease, and then a secondary increase, with markedly different slopes observed between the two ascending segments. This pattern indicates that the probe first pierced the ZP and then engaged the underlying cytoplasm in situ. Given that the indentation involved simultaneous interaction with both the ZP and cytoplasm, the slope of the second increase represents the combined elastic response of both components. To isolate the cytoplasmic contribution, we applied the Sneddon model to fit both ascending regions and subtracted the slope corresponding to the ZP. The resulting values were consistent with those reported in the literature. After conducting five measurements per oocyte, the average Young’s modulus of the cytoplasm was determined to be 1.55±0.56 kPa.

**Figure 4:**
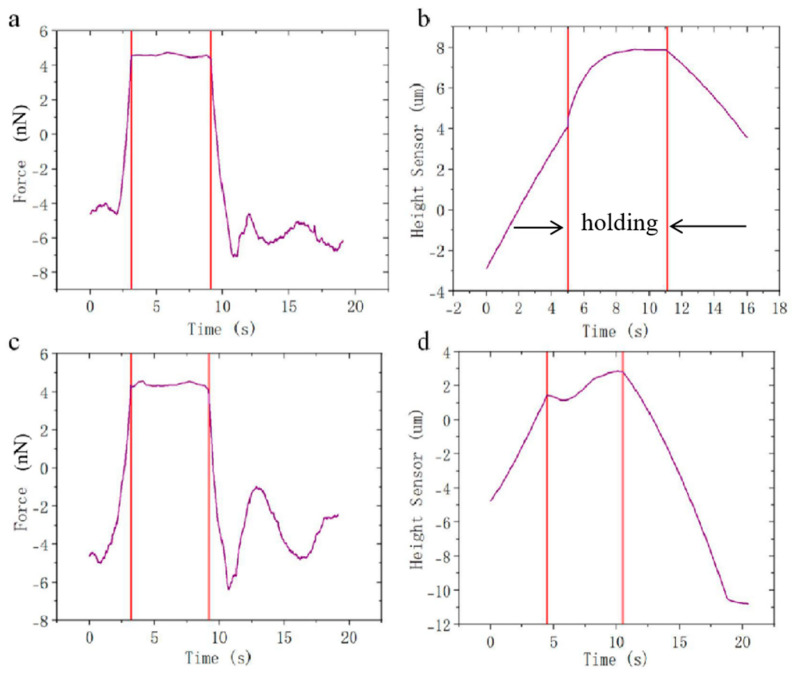
The indenting force was clamped at 5 nN for the measurement of the viscoelasticity of the ZP (**a**) and cytoplasm (**c**). The creep process of the ZP (**b**) and cytoplasm (**d**) under the fixed indenting force.

To further analyze the viscoelasticity of the ZP, we plotted the creep characteristic curve using the Surface Control Groups function of the AFM. Once the indenting force reached a predetermined value, it was kept constant and the variation in the cantilever height over time was recorded. Figure 4a illustrates the variation in indenting force over time for the measurement of ZP, with the force clamped at 5 nN and maintained for 6 s. Figure 4b shows the variation in probe height over time, providing a complete demonstration of a creep process. The probe height initially increases, with the rate of increase gradually decreasing until it approaches a stable value of 8 μm. Different from the ZP, the cytoplasm did not show a prominent process of creep due to fluid-like behavior. Figure 4c illustrates the variation in indenting force for the measurement of the cytoplasm, while Figure 4d shows the variation in probe height over time for the same process. A total of three to four sets of experimental data were obtained, among which only the most representative set is presented here.

A list of simulation parameters for the oocyte model is provided in Table 1. Among them, the Young’s modulus of the ZP in the existing literature is 10 kPa, and that of the cytoplasm is 3.16 kPa. Table 2 shows the viscosity of porcine oocyte cytoplasm at different strain rates.

### 3.2. Validation of Oocyte Model I: Oocyte Passage Through Micropipette Neck

**Figure 5:**
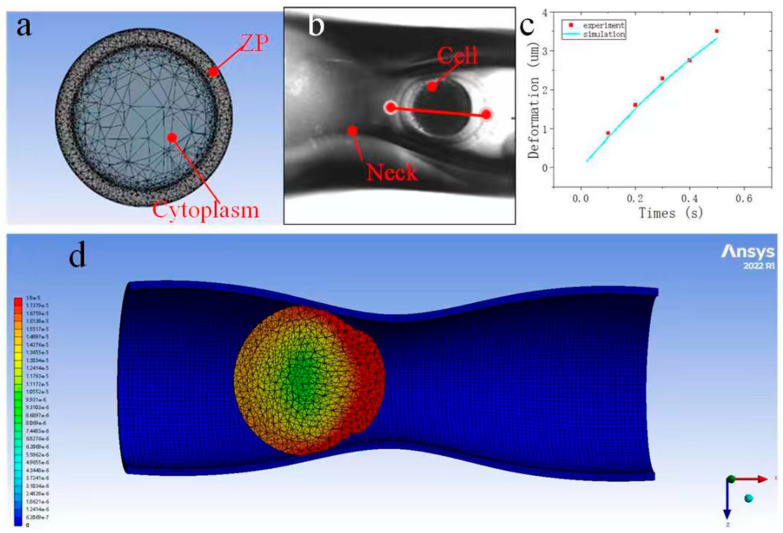
Oocyte passage through the micropipette. (**a**) Finite element model of the oocyte, constructed as a multilayered structure comprising the ZP and cytoplasm. (**b**) Experimental setup for oocyte aspiration. A custom LabVIEW-based program was developed to precisely detect the cell boundary and quantify deformation during the negative pressure-driven suction process. (**c**) Quantitative comparison between experimentally measured and simulated deformation of the oocyte as it passes through the micropipette. (**d**) Simulation output illustrating the deformation and translocation of the oocyte through the narrow-necked micropipette during aspiration.

We conducted the micropipette passage experiment using a narrow-necked micropipette with an inner diameter of 200 µm, tapered to 150 µm at 0.3 cm from the tip through heat treatment. This tapering enhanced the suction of oocytes and improved deformation retention at the neck. Once the oocyte was secured, a continuous negative pressure of 100 Pa was applied to facilitate its passage through the micropipette. To quantify cell deformation, we developed a LabVIEW program to measure the distance between any two points in the oocyte microscopic images with each pixel corresponding to 0.42 µm (Figure 5b). We marked the cell edges before and after the passage experiment (based on the positions of the anterior and posterior edges), calculating deformation by subtracting the two measurements. The reported average deformation was obtained from six independent experiments.

A three-dimensional model of the micropipette, accurately reflecting its physical dimensions, was developed using SolidWorks CAD software 2024 and subsequently imported into ANSYS for finite element simulation. This micropipette model was coupled with the layered oocyte structure previously constructed using the approach described in Section 2.3 (see Figure 5a). The interaction between the micropipette and the oocyte was defined as a face-to-face contact interface. To replicate the mechanical effect of negative pressure during aspiration, an external force calculated based on the penetration depth was applied to the oocyte in the region adjacent to the micropipette neck. The simulation was conducted over a total duration of 1 s, with 50 discrete time steps. The resulting deformation of the oocyte periphery during the suction process ranged from 8 μm to 18 μm (Figure 5d). For model validation, the simulated deformation profile was compared with empirical measurements obtained at five distinct locations along the oocyte surface, yielding an average error of approximately 5.2% (Figure 5c).

### 3.3. Validation of Oocyte Model II: Oocyte Transit Through Microfluidic Channel

**Figure 6:**
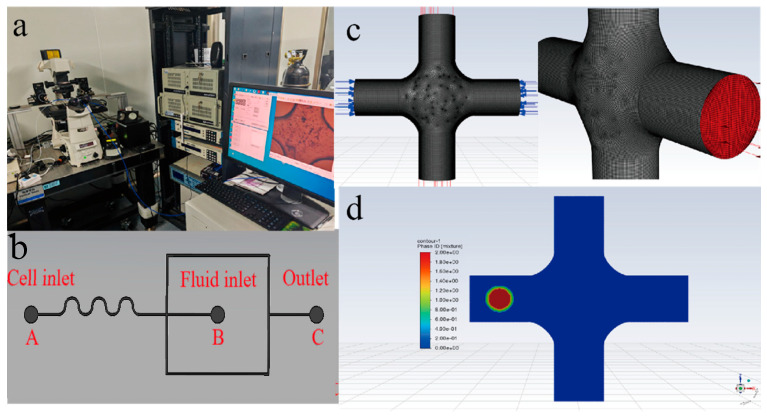
The experiment of cell transit through the microfluidic channel. (**a**) The experimental setup. (**b**) A real image of the microfluidic channel. (**c**) The hexahedral mesh of the microfluidic channel. (**d**) The three-phase flow model.

The overall experimental setup is shown in Figure 6a, which consists of an optical microscope, a microfluidic channel, a high-speed camera, a control system, and a pressure box. The pressure box controlled nitrogen gas to drive oocytes and the culture medium to transit through the microfluidic channel. The high-speed camera captured the entire process of cell deforming as they pass through the center of the microchannel. Experiments were conducted under pressures of 200, 300, 400, 500, 600, 700, and 900 hPa. The left opening (inlet A) of the microfluidic channel served as the cell inlet, while the wavy section was used to focus the cells and arrange them in sequence, ensuring they enter the center of the channel one by one. The middle opening (inlet B) served as the inlet for the culture medium. The liquids entering from both the left and middle sides converged in the center of the channel. The right opening (outlet C) served as the outlet where the liquid flowed out of the channel (Figure 6b).

The microfluidic channel was meshed into a hexahedral grid using the Cooper method in Gambit software (Figure 6c). The VOF multiphase flow model was applied, with the diameter of the oocyte set to 140 μm. The VOF (Volume of Fluid) multiphase flow model is a computational fluid dynamics (CFD) method for tracking interfaces between immiscible fluids. It uses a volume fraction function to denote the proportion of each phase in a grid cell, where a value between 0 and 1 indicates an interface, and is suitable for simulating dynamic interface changes like free-surface flows and droplet breakup. The cytoplasm had a diameter of 110 μm, while the remaining part consisted of the ZP. The channel diameter was set to 250 μm. To reduce the simulation time, the initial position of the cell was placed 500 μm away from the center of the channel (Figure 6d). The simulation time step was set to be $10^{-6}$ s, with a maximum of 16 iterations per step. The settings for the culture medium, ZP, and cytoplasm phases are listed in Table 3.

**Figure 7:**
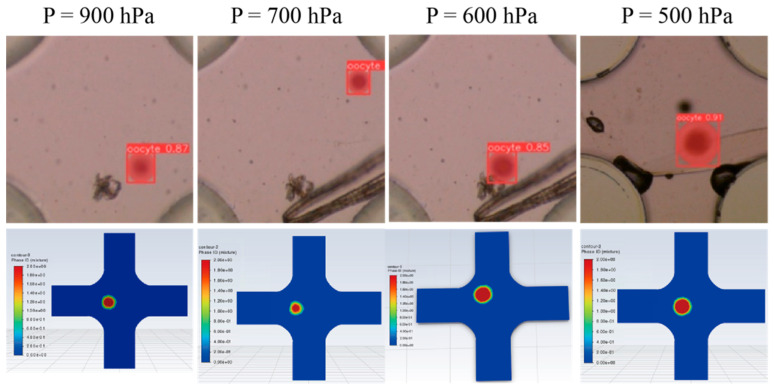
The degree of deformation of the oocytes passing through the microfluidic channel under the pressures of 900, 700, 600, and 500 hPa in the physical experiments (**top**) and simulations (**bottom**).

The degree of oocyte deformation was measured by the ratio of the major to minor axes of the ellipse formed after the cell’s deformation. This ratio was obtained using instance segmentation with the Yolov8 model. As the pressure increases, the requirements for the frame rate of the camera become higher, and our current setting can reach up to 900 hPa. We collected seven sets of data by gradually reducing the pressure from 900 hPa to 200 hPa. Figure 7 presents the experimental and simulation results at pressures of 900, 700, 600, and 500 hPa, with detailed values listed in Table 4. The experimental results showed strong agreement with the simulation, exhibiting a deviation of less than 4% under consistent spatial and temporal scales.

## 4. Discussion

In this study, we developed two distinct viscoelastic models of oocytes to accurately simulate their mechanical behavior under different interaction scenarios. The first model employs a layered solid FE model to represent oocytes interacting with manipulation tools, while the second utilizes a three-phase flow model to describe oocyte interactions solely with fluid environments. By modeling the oocyte as a layered structure with distinct mechanical properties for the ZP and cytoplasm, our model captures the heterogeneous nature of oocytes more effectively than homogeneous models. Interactions with tools involve forces like compression, squeezing, and punction, which are best described by solid mechanics. The three-phase flow model was designed to simulate oocyte deformation within microfluidic channels. Fluid dynamics play a critical role in oocyte behavior in microfluidic devices. A multiphase flow model accounts for fluid–structure interactions, such as shear stress at fluid junctions or pressure gradients within microchannels, which solid models cannot simulate. Both models were rigorously validated through experiments—narrow-necked micropipette tests for the layered FE model and microchannel experiments for the three-phase flow model. The close agreement between simulation and experimental results implies the robustness and applicability of our modeling approaches.

Unlike previous studies that relied on optimization algorithms to estimate mechanical properties, our approach directly incorporates AFM-derived parameters into the models. This significantly simplifies the parameter identification process, making it more efficient and accessible for practical applications. We first measured the Young’s modulus of the ZP, obtaining a value of 7 kPa, which is slightly lower than the typical values of 10 kPa from [2,30]. Using a specialized measurement method, we also measured the Young’s modulus of the cytoplasm, obtaining a value of 1.55 kPa, which is also consistent with the study in [31]. We then derived the viscoelastic properties of the oocytes by calculating the Young’s modulus and shear modulus at each sampling point during the creep process, utilizing the “Shear Data—Viscoelastic” command in ANSYS software.

We validated the effectiveness of the layered FE model using a fine-necked micropipette experiment. As the oocyte passed through the narrow part, it interacted with the tube wall, deforming under the compression exerted by the wall. To replicate this process in simulation, we constructed a fine neck with identical dimensions in ANSYS and the oocyte model. The simulation results showed that the deformation of the cell periphery deviated by less than 5% from the experimental measurements. We validated the multiphase flow model using a microchannel experiment. At fluid junctions within the microchannel, the oocyte experienced shear stress. We simulated this deformation process under varying pressure conditions and quantified the deformation using the aspect ratio of the oocyte’s major and minor axes. The error between the experimental and simulated results remained within 4%, demonstrating the reliability of the multiphase flow model.

Despite the strengths of our study, several limitations warrant consideration. First, our models assume a spherical geometry for oocytes, which may not fully capture the shapes and asymmetries observed in vivo. Incorporating detailed geometries obtained through advanced imaging techniques, such as confocal microscopy or 3D reconstruction, into the FE and multiphase flow models could improve the accuracy of simulations. Second, while the three-phase flow model accounts for fluidic stimuli, it does not extensively address dynamic interactions, such as oscillatory flows or varying shear rates. Future simulations under dynamic conditions, such as time-varying pressures, would better replicate physiological environments and provide a more robust assessment of the models’ performance. Finally, our models were validated under specific experimental conditions. Further validation across a broader range of experimental setups would enhance the applicability and reliability of the models.

## 5. Conclusions

This study contributes two novel viscoelastic models for simulating the mechanical behavior of oocytes under distinct interaction scenarios. The first model is a layered FE structure designed to capture the interaction between oocytes and manipulation tools, while the second is a three-phase flow model that describes the deformation of oocytes within fluid environments, reflecting their complex fluid–structure interactions. This dual modeling approach bridges the gap between solid and fluid mechanics in understanding oocyte behavior. We modeled oocytes as a layered structure, considering the distinct mechanical properties of the ZP and cytoplasm. The material parameters were directly obtained through AFM measurements, bypassing the limitations of optimization-based estimations. Additionally, the three-phase flow model offers a novel approach by representing oocytes as “liquid in liquid” systems, which accurately reflects their dynamic internal structure and interaction with the culture medium. The FE model accurately simulated the deformation of oocytes passing through narrow-necked micropipettes, with a simulation error of approximately 5.2% compared to physical experiments. Similarly, the three-phase flow model effectively replicated oocyte deformation within microfluidic channels, achieving a close agreement with experimental data under varying pressures, with an error margin of less than 4%. Future simulations under dynamic conditions, such as time-varying pressures, oscillatory flows, or varying shear rates, would better replicate physiological environments and provide a more robust assessment of the models’ performance. In conclusion, this study offers a comprehensive framework for simulating oocyte mechanics, providing valuable insights into their behavior across different conditions.

## Figures and Tables

**Table 1:** A list of simulation parameters for the oocyte model.

	Parameter	Simulation
ZP	Radius Young’s Modulus Poisson’s Ratio	15 um 7 kPa 0.5
Cytoplasm	Radius Young’s Modulus Poisson’s Ratio	75 um 1.55 kPa 0.5
Pipette	Inner Diameter External Diameter Young’s Modulus	90 µm 108 µm 5.5 × 10^7^ kPa
Contact mode	ZP and Cytoplasm Pipette and ZP	Bonded Frictional Friction Coefficient 0.2

**Table 2:** The viscosity of porcine oocyte cytoplasm under different strain rates.

Strain Rate (1/s)	Stress (Pa)	Viscosity (Pa s)
0.00172	0.00213	969.1
0.00183	0.00214	940
0.00456	0.00217	467.8
0.006	0.00225	365.4
0.0107	0.00231	215
0.0119	0.00221	186.1
0.021	0.00256	125.8
0.0222	0.00274	124.7
0.0229	0.00289	121.9
0.0238	0.00245	102.8
0.0524	0.00462	44.1

**Table 3:** The settings for the culture medium, ZP, and cytoplasm phases.

	Culture Medium Phase	ZP Phase	Cytoplasm Phase
Density (kg/m^3^)	1008	1047	1020
Viscosity (Pa s)	$1.66\times 10^{-3}$		
Zero-shear viscosity (Pa s)		4617.9	359.89
Infinite-shear viscosity (Pa s)		3.371	1.368

**Table 4:** The ratio of the major to minor axes of the ellipse formed after the deformation of the cell. Unit: hPa.

	900	700	600	500	400	300	200
Experiment	0.908	0.911	0.912	0.921	0.931	0.928	0.974
Simulation	0.922	0.931	0.934	0.942	0.958	0.967	0.979

## Data Availability

The data that support the findings of this study are available on request.
